# MicroRNA and Heart Failure

**DOI:** 10.3390/ijms17040502

**Published:** 2016-04-06

**Authors:** Lee Lee Wong, Juan Wang, Oi Wah Liew, Arthur Mark Richards, Yei-Tsung Chen

**Affiliations:** 1 Cardiovascular Research Institute, Department of Medicine, Yong Loo Lin School of Medicine, National University of Singapore, #08-01, MD6 Centre for Translational Medicine, 14 Medical Drive, Singapore 117599, Singapore; mdcwll@nus.edu.sg (L.L.W.); mdcwaju@nus.edu.sg (J.W.); mdclow@nus.edu.sg (O.W.L.); arthur_mark_richards@nuhs.edu.sg (A.M.R.); 2 Cardiac Department, National University Health System, Tower Block Level 9, 1E Kent Ridge Road, Singapore 119228, Singapore; 3 Christchurch Heart Institute, Department of Medicine, University of Otago, PO Box 4345, Christchurch 8014, New Zealand

**Keywords:** microRNA, cardiovascular, heart failure, neurohormone

## Abstract

Heart failure (HF) imposes significant economic and public health burdens upon modern society. It is known that disturbances in neurohormonal status play an important role in the pathogenesis of HF. Therapeutics that antagonize selected neurohormonal pathways, specifically the renin-angiotensin-aldosterone and sympathetic nervous systems, have significantly improved patient outcomes in HF. Nevertheless, mortality remains high with about 50% of HF patients dying within five years of diagnosis thus mandating ongoing efforts to improve HF management. The discovery of short noncoding microRNAs (miRNAs) and our increasing understanding of their functions, has presented potential therapeutic applications in complex diseases, including HF. Results from several genome-wide miRNA studies have identified miRNAs differentially expressed in HF cohorts suggesting their possible involvement in the pathogenesis of HF and their potential as both biomarkers and as therapeutic targets. Unravelling the functional relevance of miRNAs within pathogenic pathways is a major challenge in cardiovascular research. In this article, we provide an overview of the role of miRNAs in the cardiovascular system. We highlight several HF-related miRNAs reported from selected cohorts and review their putative roles in neurohormonal signaling.

## 1. Introduction

The cardiovascular system (CVS), comprising cardiac, vascular and hematopoietic components, is one of the earliest organ systems to develop in vertebrates. The CVS transports oxygen and nutrients to all organs and removes metabolic waste via the capillary and venous system. In addition, it transports endocrine factors and immune cells throughout the body. In mammals, thermoregulation is a key function of the CVS whereby appropriate vasodilation and vasoconstriction of peripheral blood vessels adjusts blood flow to the skin to maintain core body temperature. Cardiovascular pressure/volume homeostasis depends on neurohormonal systems including the renin-angiotensin-aldosterone (RAAS) and sympathetic nervous (SNS) systems together with the cardiac natriuretic peptides. In the context of cardiac injury inappropriate activation of RAAS and SNS induces deleterious haemodynamic, renal and myocardial trophic effects which accelerate progression to frank HF with its attendant high morbidity and mortality. Therapeutics that target these pathways have proven to be effective in ameliorating the symptoms and prolonging the lifespan of HF patients [1]. With respect to other neurohormonal pathways, the natriuretic peptide hormones (atrial natriuretic peptide (ANP) and B type natriuretic peptide (BNP), and adrenomedullin (ADM)) are examples of hormonal and paracrine effectors which offer beneficial compensatory responses generally opposed to the actions of the RAAS and SNS. They are powerful diagnostic and prognostic biomarkers. They may also provide the foundation for the next generation of therapeutics based on their cardioprotective effects [2,3,4,5]. This is currently best exemplified by the introduction of LCZ 696, a drug which both blocks the angiotensin 2 type 1 receptor and inhibits neprilysin, the dominant enzyme for degradation of the natriuretic peptides. This dual manipulation of neurohormonal pathways has proven to be a major advance in HF therapy reducing all-cause mortality and recurrent hospital admissions in chronic HF by approximately 20% [6].

The discovery of microRNA (miRNA), highly conserved small non-coding RNAs, has opened up new avenues in the study of regulation of gene expression. It is now known that miRNAs are widely involved in gene regulation from normal development through to the pathogenesis of disease. Evidence from experimental platforms points to potential applications of miRNAs for diagnostic and therapeutic purposes [7,8]. Herein, we provide an overview of miRNA functions in cardiovascular development and disease. We discuss the use of miRNA target prediction algorithms applied to published HF-related miRNA entities to identify putative target genes in the neurohormonal networks. Finally, we consider their possible therapeutic applications in cardiovascular disease.

## 2. MicroRNA Discovery and Biogenesis

**Figure 1:**
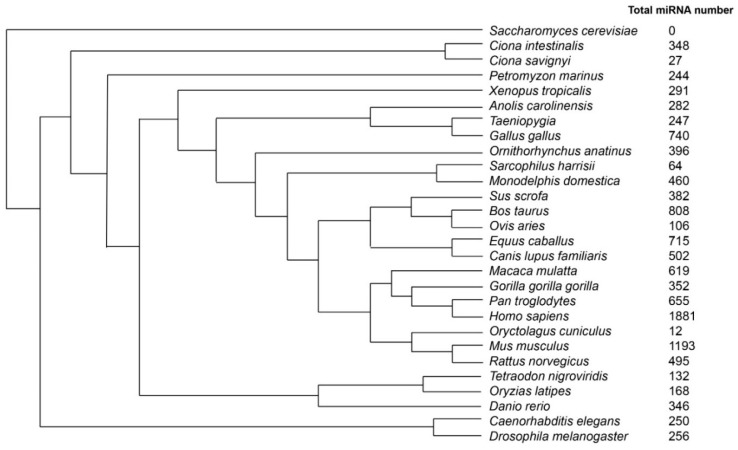
Summary of the total number of miRNA [14] identified in different species obtained from the latest version of miRBase v 21 database. The phylogenetic tree is generated according to the species tree obtained from Ensembl website [15].

In 1993, two groups simultaneously published the discovery of the first noncoding miRNA, lin-4, in *Caenorhabditis elegans* (*C. elegans*) and demonstrated the interaction of lin-4 transcripts with the complementary sequence present in the 3′ untranslated region (3′UTR) of lin-14 mRNA [9,10]. The authors further demonstrated that the complementary RNA-RNA interaction between lin-4 transcript and lin-14 mRNA 3′UTR is essential for the repression of synthesis of the lin-14 protein, suggesting an inhibitory effect of lin-4 on translation of lin-14. Subsequently, investigators have identified numerous miRNAs conservatively expressed across eukaryotes [11]. Figure 1 shows the number of miRNAs found in various species listed in the miRBase v.21 [12]. As the most well studied species, the greatest number of miRNAs has been found in the human genome. It is estimated that miRNAs participate in modulating the expression of more than 60% of protein-coding genes [13].

**Figure 2:**
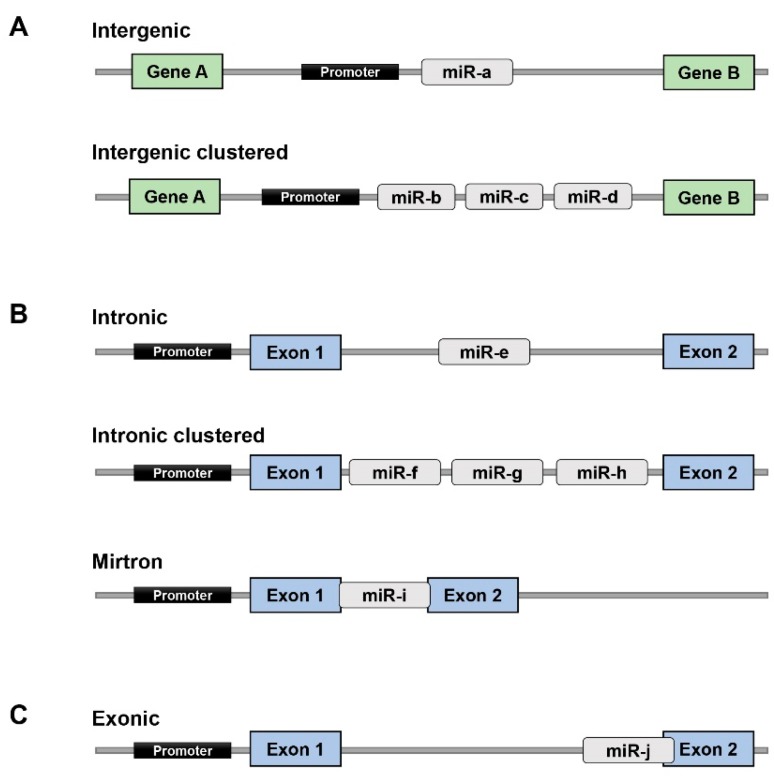
Genomic location of miRNAs. (**A**) Intergenic miRNAs are found in genomic regions between genes. They may be present as a single miRNA (miR) gene or a cluster of miRNA genes; (**B**) Intronic miRNAs are found in the introns of annotated genes. Like intergenic miRNAs, intronic miRNAs may exist in the single or clustered format and can also overlap with exons; (**C**) Exonic miRNAs often span across an exon and an intron of a noncoding gene.

miRNAs can be classified as intergenic, intronic or exonic miRNAs depending on their genomic location and gene structure [16]. Almost half of known miRNA genes are located in the intergenic region. These may exist either as a single gene or a cluster of genes under the control of their own promoters (Figure 2A) [17,18]. Intronic miRNAs, are located in the introns of annotated genes, including both coding and non-coding genes. These intronic miRNA genes could be co-transcribed with their host genes or by their own miRNA-specific promoter (Figure 2B). Exonic miRNAs are found overlapping across an exon and an intron of noncoding genes. This class of miRNAs is rare compared with the other two types (Figure 2C) [16,19].

**Figure 3:**
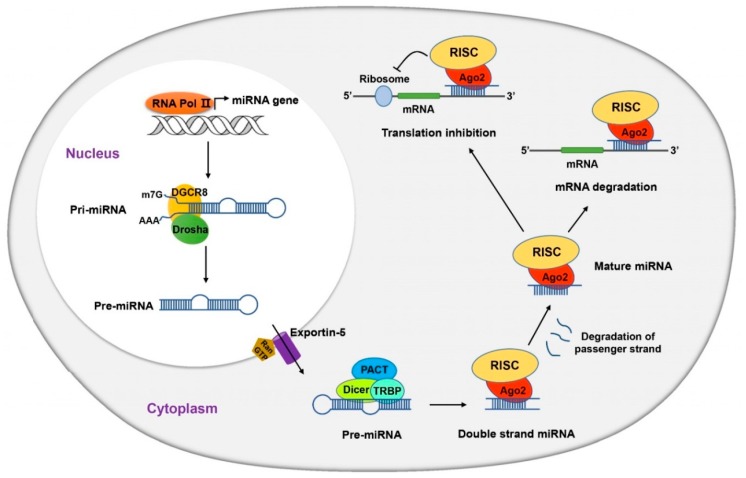
Biogenesis of miRNA. Please refer to the main text for details. RNA Pol II: RNA Polymerase II; Pri-miRNA: Primary-miRNA; Pre-miRNA: Precursor-miRNA; DGCR8: DiGeorge syndrome critical region gene 8; Drosha: Ribonuclease Type III; Dicer: RNase III endonuclease; PACT: protein activator of the interferon induced protein kinase; TRBP: TAR RNA binding protein; RISC: RNA induced silencing complex; Ago2: Argonaute 2.

miRNAs modulate gene expression through several mechanisms including inhibition of translation, repression of mRNA expression, initiation of mRNA degradation, mRNA de-adenylation and mRNA sequestration [20,21,22]. As illustrated in Figure 3, mature miRNA formation originates from a long primary miRNA (pri-miRNA), several hundred nucleotides or kilobases in length, which is transcribed by RNA polymerase II. The pri-miRNA has a characteristic stem-loop structure with a 5′-cap (7MGpppG) and 3′-end poly-adenylated tail (Poly-A) that can be recognized and further cleaved by ribonuclease III (RNase III) endonuclease, Drosha, along with its essential cofactor, DiGeorge syndrome critical region gene 8 (DGCR8) [19,23]. The approximately 70-nucleotide precursor miRNA (pre-miRNA) with a stem-loop structure, so formed, is then transported from the nucleus to the cytoplasm by nuclear export factors, Exportin-5 and Ran-GTP [19]. In the cytoplasm, the pre-miRNA is further cleaved into an approximately 22-nucleotide double-stranded miRNA by another RNase III endonuclease, Dicer, and its associated double-stranded RNA binding protein, TAR RNA binding protein (TRBP) and protein activator of the interferon induced protein kinase (PACT). Subsequently, the small RNA duplex is loaded onto particular Argonaute (AGO) proteins, such as AGO2, along with other cofactors to form the RNA induced silencing complex (RISC). Mature miRNA (also known as guide RNA strand) is formed when it is separated from the passenger RNA strand and the released passenger strand is rapidly degraded [24]. The RISC assembly carrying the single-stranded guide miRNA attaches to its target nucleotide segment by Watson-Crick sequence complementarity between the miRNA and the 3′UTR of the target mRNA transcript. This triggers the degradation/deadenylation of the target mRNA or repression of translational machinery [21,22].

## 3. Landmark MicroRNA Studies in Cardiovascular Field

Mounting evidence indicates miRNAs are involved in almost all developmental, physiological, and pathological processes, including those occurring in the CVS [25,26]. Advances in gene targeting technologies have revealed the vital roles of miRNAs in cardiovascular development and pathology and in maintaining normal physiological functions [27,28,29,30,31,32]. These landmark studies have established the critical roles of miRNAs in embryonic and postnatal cardiac development as well as in normal functioning of the healthy adult heart. Cardiac-specific deletion of Dicer leads to impaired miRNA biogenesis. miRNAs are not only essential for cardiac development and normal heart functioning, but are also influence cardiac remodeling in cardiovascular disease. These early pivotal studies established and validated approaches for deciphering miRNA networks and defining how these are superimposed on transcriptional and signaling pathways. This has provided our current technical toolkit for identifying and validating specific miRNAs and their mRNA targets. Such knowledge has deepened our understanding of how cardiac cell fate and cardiac morphogenesis are determined. This in turn provides opportunities for developing therapeutic approaches to ameliorate or reverse cardiovascular disease.

### 3.1. Dicer in Cardiac Development

Disruption of key factors in miRNA biogenesis has defined the importance of miRNAs in embryonic development. Experimental ablation of Dicer causes abnormalities in body plan configuration before gastrulation, leading to early embryonic lethality. Likewise, Ago2-null animals bear multiple abnormalities in neural and cardiac development and manifest embryonic lethality [33,34,35]. Numerous transgenic studies have dissected the roles of Dicer and Ago2 at different developmental stages and in different tissues in adult animals. The functions of Dicer in cardiac development in particular have been addressed in floxed Dicer mouse lines and mouse lines that express Cre recombinase driven by different developmental stage-specific cardiac promoters. The role of Dicer in early cardiac development has been assessed in Nkx2.5-Cre and floxed Dicer transgenic mice. Cardiac-specific deletion of Dicer causes abnormalities in formation of both ventricular myocardium and of the cardiac outflow tract together with deficient chamber septation. These effects culminate in embryonic lethality [36]. The role of Dicer in later development has been examined in transgenic mice engineered to express the alpha myosin heavy chain (α-HMC) promoter-driven Cre recombinase and floxed Dicer. Cardiac-specific deletion of Dicer in the post-mitotic stage did not affect chamber septation but led to a significant decrease in cardiac contractility, severe dilated cardiomyopathy, HF, and rapid progression to death within four days after birth [37]. To further elucidate the role of miRNAs in the post-developmental and adult heart, a tamoxifen-induced α-HMC Cre/floxed Dicer transgenic system was applied in young and adult mice. Interestingly, selective deletion of Dicer in cardiac myocytes of three-week old mice triggered premature death within one week with manifestation of mild ventricular remodeling and dramatic atrial enlargement. In eight-week old adult mice, myocardium specific Dicer-knockdown resulted in reduced expression of cardiomyocyte-specific miRNAs in association with morphological and functional changes including cardiac hypertrophy, myocyte disarray, fibrotic lesions and reduced cardiac contractility [38]. Decline in cardiac function in Dicer-ablated adult hearts was associated with re-activation of the fetal gene program.

### 3.2. Specific miRNAs in Cardiac Development

#### 3.2.1. miR-1 and the miR-133a Superfamily

The functions of individual miRNAs at different cardiac developmental stages have been actively explored and characterized in animal models. Two muscle specific miRNAs: miR-1 and miR-133a, are functionally cooperative in promoting mesoderm differentiation in embryonic stem (ES) cells whilst repressing ectodermal and endodermal differentiation [39,40]. miR-1 affects cardiomyocyte growth by negatively regulating expression of calmodulin and calmodulin-dependent nuclear factor in activated T cells (NFAT) signaling. In addition to targeting calmodulin, miR-1 also targets the 3′UTRs of several gene transcripts important in cardiomyocyte growth. These include myocyte enhancer factor 2A (Mef2a) and GATA binding protein 4 (Gata4). miR-1 also targets key cardiac-specific transcription factors, notch ligand, Delta like 1 (Dll-1), Iroquois-class homeodomain protein (Irx-5) and Heart and neural crest derivatives expressed 2 (Hand2). A further target gene is potassium voltage-gated channel subfamily D member 2 (Kcnd2) [41,42,43]. Loss-of-function miR-133a mutants exhibit increased proliferation of cardiomyocytes and up-regulation of smooth muscle cell-specific genes. miR-133 regulates cardiomyocyte proliferation by repressing cyclin D2 and serum response factor (Srf) [44]. Together with miR-1 and miR-133, miR-27 is also expressed during early cardiogenesis, and controls venous differentiation and endothelial tip cell fate via modulation of Mef2c expression [45,46].

#### 3.2.2. miR-208 and the miR-499 Superfamily

miR-208a, miR-208b and miR-499 are encoded by the introns of three muscle-specific myosin genes Myh-6, Myh-7, and Myh-7b, respectively. These three miRNAs are differentially expressed in a spatiotemporal fashion in embryonic and adult heart and their actions demonstrate the participation of intronic miRNAs in processes associated with their host gene functions. Cardiac-specific overexpression of miR-208a is sufficient to induce hypertrophic growth and arrhythmias in mice. Depletion of miR-208a in a mouse knockout model causes defective cardiac conduction with attenuated expression of homeodomain-only protein [47] and connexin 40 (Cx40) [48]. Overexpression of miR-208a and miR-208b causes post-transcriptional repression of genes including TNF receptor-associated protein 1 (Trap1) and myostatin as well as the transcription factor Gata4. This suggests the miR-208 family is involved in the formation of cardiac myosin and transcriptional activation of genes that contain the serum response factor-dependent promoter region. Interestingly, miR-499 shares common mRNA targets with miR-208, and some of these target genes also overlap with miR-1 targets, suggesting associations with cardiac differentiation [49]. In corroboration, ablation of miR-499 in mouse and human ESC culture blocks cardiomyocyte differentiation, while over-expression of miR-499 promotes the early formation of clusters of beating embryoid bodies, indicating enhancement of myogenic differentiation [50].

#### 3.2.3. miR-15 Family

The miR-15 family comprises a cluster of miRNAs (miR-15a, miR-15b, miR-16-1, miR-16-2, miR-195 and miRA-497) that share a common seed region (AGCAGC) in the 5′ end of their cognate mature miRNAs. Upregulation of the miR-15 family was observed during postnatal cardiac development, particularly miR-195 which is the most abundant species among the six members. Further analyses focused on miR-195 demonstrated its regulatory effects on key regulators of binucleation and cell cycle withdrawal, including cell cycle checkpoint kinase 1 (Chek1), cell division cycle 2 homolog A (Cdc2a), Baculoviral IAP repeat-containing protein 5 (Birc5), nucleolar and spindle associated protein 1 (Nusap1), and sperm-associated antigen 5 (Spag5) [51]. Anti-miR mediated postnatal knock-down of the miR-15 family increases the number of cardiomyocytes undergoing mitosis, revealing the regulatory role of the miR-15 family in postnatal cardiac development. [51]. The miR-15 family is involved in the modulation of neonatal heart regeneration by attenuating the proliferation of cardiomyocytes [52].

### 3.3. miRNAs in Vascular Integrity

miRNAs act in angiogenesis and vessel maturation and also regulate vascular and endothelial cell function. Gene targeting in mouse and zebrafish models have elucidated the roles of endothelial cell-specific miR-126 in vascular integrity, endothelial cell proliferation and migration [53,54,55]. Mice lacking miR-126 exhibit defective angiogenesis and have fragile and leaky vessels while knock-down of miR-126 in zebrafish is accompanied by total embryonic lethality arising from loss of vascular integrity and haemorrhaging. miR-126 enhances MAP kinase signaling and PI3K signaling by inhibiting the negative regulators sprouty-related protein 1 (Spred1), and phosphoinositide-3-kinase-regulatory subunit 2a (PI3KR2a), respectively [55]. miR-126 also down-regulates the expression of vascular cell adhesion molecule 1 (VCAM1) and interrupts the tumor necrosis factor alpha (TNFα) signaling cascade in endothelial cells [56].

The miR-17/92 cluster (including miR-17, miR-18a, miR-19a, miR-20a, miR-92a, and miR-92b) originally identified as critical for neovascularization in tumors, is predominantly expressed in endothelial cells [57,58]. Several lines of evidence demonstrate that this cluster targets the expression of proangiogenic genes and hence regulates angiogenesis. MiR-17, miR-18a, miR-19a, and miR-20a have anti-angiogenic effects as revealed by increased blood vessel and endothelial cell sprout formation after introduction of specific antagomirs. The predominant miRNA in this cluster, miR-17 exerts its antiangiogenic effects by regulating the expression of Janus kinase 1 [57]. Similarly, miR-92a targets integrin subunit alpha 5 (Itgα5) in endothelial cells causing attenuation of endothelial sprout formation. Antagonism of miR-92a improves blood vessel growth and functional recovery of damaged tissue after experimental myocardial infarction in mice [59]. The role of miRNAs in endothelial function and vascular-associated disease was recently reviewed by Santulli [60].

## 4. Heart Failure

HF is a complex syndrome defined by a cardiac output inadequate to meet the metabolic demands of body tissues. HF results from a wide range of congenital and acquired cardiovascular or metabolic diseases leading to structural and functional impairment of the heart [61]. Globally, HF is the leading cause of hospitalization in adults over the age of 65 years [62]. One in five adults currently aged 40 will develop HF in their remaining lifetime. Despite advances in the treatment of HF, morbidity and mortality (~50% at five years) remains high and constitutes a significant economic and public health burden [63]. It is estimated that, in the United States, the prevalence of HF will surge by 46% from 2012 to 2030 to more than 8 million. Treatment costs are estimated to increase from $31 billion in 2012 to $70 billion in 2030 [64].

HF can also be defined on the basis of ventricular abnormalities that can occur in the right or left ventricle although impairment of left ventricular myocardial function is predominant along with systemic congestion. Patients with HF may also be classified on the basis of their left ventricular ejection fraction (LVEF) which may be reduced compared with normal (HF with reduced ejection fraction or HFREF) or preserved relative to normal (HF with preserved ejection fraction or HFPEF) [61]. In this section, we provide an overview of the current understanding of the etiology of heart failure, the involvement of neurohormonal signaling in cardiovascular homeostasis, and also discuss existing diagnostic tools and treatment options in HF. This background information will provide the framework for better understanding of the putative involvement of miRNAs in the pathogenesis of HF and their potential as markers and/or therapeutic targets in HF.

### 4.1. Etiology of Heart Failure

Epidemiological studies have revealed numerous risks and causative factors for HF (Table 1) [61,65,66,67]. Studies of the medical records of more than 300,000 enrollees with the Health Management Organization, Kaiser Permanente confirmed the association of five major antecedent factors for HF, including coronary artery disease, hypertension, diabetes mellitus, atrial fibrillation and valvular heart disease [65]. The Framingham heart study further revealed that higher serum creatinine, lower ratio of forced expiratory volume in 1 s to forced vital capacity (FEV1:FVC ratio; pulmonary), and lower hemoglobin concentrations are associated with increased risk of HF [66]. Lifestyle factors such as smoking, obesity, alcohol consumption and lack of exercise also contribute to risk of HF often via acceleration of atherosclerotic processes or the development of diabetes and hypertension [67].

There are well-documented differences between HFREF and HFPEF. Patients with HFPEF are generally older, more often female, less likely to have coronary artery disease (CAD), and more likely to have hypertension [68,69,70]. This suggests that different mechanisms may underlie these two major phenotypes of HF.

### 4.2. Neurohormonal Signaling and Heart Failure

The term “neurohormone” originally referred to hormones secreted by neuroendocrine cells. In the cardiovascular biomedical arena, this term has become broadly applied to include true neurohormones (NH) as well as the entire range of endocrine, paracrine and autocrine factors which in aggregate influence cardiac, vascular, renal and adrenal structure and function in the context of cardiac injury and HF. They can be crudely categorized into two groups; one is characterized by inappropriate activation and deleterious effects, and the other by beneficial compensatory effects. Adverse actors include the effectors of the RAAS (renin, angiotensin II and aldosterone) and the SNS (epinephrine, norepinephrine). This group also includes arginine-vasopressin and endothelin. These panels of NH are vasoconstrictors with anti-natriuretic, inotropic, hypertrophic and fibrotic actions. In the normal state, blood pressure is sensed by arterial baroreceptors that mediate activation of the SNS and norepinephrine secretion. Circulating catecholamines, in concert with elevated SNS neural traffic, stimulates α1 and β adrenergic receptors producing vasoconstriction, cardiac inotropism and activation of the RAAS [71]. This cluster of NH enhances renal retention of sodium and enhances ventricular contractility, cardiac output and blood pressure in an effort to meet the metabolic demands of the body. Evolutionarily, the SNS works together with RAAS as survival mechanisms to counter depletion of circulating volume or oxygen delivery secondary to blood loss or other causes of volume depletion such as gastrointestinal losses. However, in HF this array of actions is deleterious and inexorably sustains an excess circulating volume and elevated cardiac afterload whilst simultaneously promoting cardiac hypertrophy and fibrosis and accelerated cardiomyocyte apoptosis. The latter actions mediate adverse left ventricular remodeling which in turn accelerates decline in ventricular function. This cycle leads to the high rate of decompensated HF and death that characterizes this syndrome. Conversely an opposing group of NH comprising the cardiac natriuretic peptides (NP; including ANP [2], BNP and C-type natriuretic peptide (CNP)), ADM and the Urocortins exert natriuretic, diuretic, vasodilator, anti-hypertrophic, and anti-fibrotic actions [72,73,74]. This cluster of NH is cardio-protective and ameliorates the duress imposed by cardiac injury or pressure overload.

In HF, complex neurohormonal responses involving both beneficial and deleterious components are activated in response to compromised cardiac function caused by acute or chronic cardiac injury. Table 2 summarizes the functions of NH in normal and diseased states.

### 4.3. Diagnosis of Heart Failure

There is no single diagnostic test for HF. It is first and foremost a clinical syndrome diagnosed initially by recognition of suggestive symptoms and signs with corroboration sought from imaging such as echocardiogram to confirm an associated left ventricular structural and functional abnormality [61,85]. The electrocardiogram (ECG) and chest X-ray are useful ancillary investigations. In some cases, cardiac computerized tomography (CT), magnetic resonance imaging (MRI), coronary angiography, or myocardial biopsy to examine the functional and structural abnormalities of the heart and to evaluate coronary artery functional integrity are performed to clarify etiology. Increased understanding of HF has provided impetus to the development of diagnostic and prognostic biomarkers for HF.

Circulating levels of many NH including norepinephrine, renin, angiotensin, aldosterone, arginine-vasopressin and endothelin are both elevated and prognostic in HF. Plasma NP concentrations (ANP, MR-proANP, BNP and NT-proBNP) reflect ventricular function and also have prognostic value in HF [86,87]. Plasma levels of cardiac specific structural proteins, such as troponin T and troponin I, or the key component of cell membrane, lectin-like oxidized low-density lipoproteins receptor-1 (LOX-1), in circulation also reflect the severity of cardiac injury and/or dysfunction [88,89,90]. The inflammatory factor, interleukin 6 (IL-6), tumor necrosis factor alpha (TNFα) and factors that involved in fibrosis and hypertrophy, such as c-reactive protein (CRP), matrix metalloproteinases (MMP), galectin-3, soluble ST2 (interleukin 1 receptor), were suggested to be indicators of the cardiac remodeling process [91,92]. To date, BNP and NT-proBNP are the established diagnostic and prognostic biomarkers for HF whilst troponins have been applied in ACS, and these biomarkers are endorsed by all authoritative management guidelines clinical management of HF and ACS [61,93,94,95,96]. Table 3 lists, by no means exhaustively, the HF biomarkers gleaned from numerous published reports.

The use of BNP/NT-proBNP plasma levels for diagnosis and risk prediction of recurrent cardiac decompensation and death has brought significant improvement in HF disease management and treatment. However, several partially confounding factors, such as age, renal function, obesity and atrial fibrillation limit the accuracy of NT-proBNP and BNP as diagnostic and prognostic tests [97,98], thus, the identification of additional biomarkers for HF constitute an unmet need for further improvement in the accuracy of HF diagnosis and guidance of treatment. Several research groups, including ours are devoted to identify therapeutic targets and further delineate the various HF subtypes for improved diagnosis and biomarker-assisted care. Currently, the clinical introduction of testing for troponins and BNP/NT-proBNP has fueled interest in the utilization of other putative HF biomarkers listed under the categories of neurohormonal activation, myocardial overload, cardiac injury and cardiac remodeling in Table 3. For clinical utility candidate biomarkers must provide diagnostic, prognostic and/or other information to guide HF management, in addition to that available from current methods.

### 4.4. Treatment of Heart Failure

Medicines that block RAAS and SNS are the mandated evidence-based therapies for HF. Most HF patients also receive diuretics. In cases with defined causes for HF such as valvular disease, coronary artery disease, or risk of sudden death associated with given degrees of ventricular impairment, surgical interventions or device implantation may be recommended in addition to full pharmacotherapy. Currently, effective HF medications target over-activated adverse NH signaling. Medication for HF treatment may be classified according to their drug targets: (1) Drugs that oppose the RAAS include angiotensin 2 type 1 receptor blockers (ARBs), angiotensin converting enzyme inhibitors (ACEIs) and mineralocorticoid antagonists (MRAs); (2) β-Adrenergic receptor blockers block catecholamine binding to adrenoceptors; (3) Drugs that are used to change the vessel tone or blood pressure/volume include an array of vasodilators or diuretics; (4) Medicines that improve ventricular contraction, *i.e*., positive inotropes include digoxin and other agents [99,100].

Current evidence of drug efficacy is limited to HFREF. HFPEF patients do not obtain similar clinical benefits from ACE inhibition, angiotensin receptor blockade or β blockade as patients with HFREF [101,102]. Several clinical trials have shown that mineralocorticoid receptor antagonist (MRA) treatment improves outcomes in systolic HF [103]. The role of the MRA, spironolactone, in treatment of HFPEF was tested in the Treatment of Preserved Cardiac Function Heart Failure with an Aldosterone Antagonist (TOPCAT) trial [104]. Overall results were negative. However, a later post-hoc analysis revealed spironolactone benefited patients enrolled in the Americas but not in Russia and Georgia, the former having a much higher-risk profile compared with the latter [105]. Interestingly, analysis of two other large HFPEF trials—the CHARM-Preserved trial with candesartan and the I-PRESERVE trial with Irbesartan—also showed similar regional disparities in outcomes between the Americas and Eastern Europe even after adjustments for key prognostic baseline variables [106]. Clearly, further investigations are needed to better define the risks and benefits of administering this neurohormonal antagonist in HFPEF patients for which evidence-based therapies are lacking and the striking geographical disparities in outcomes possibly arising from regional differences in existing comorbidities and clinical practice patterns need to be carefully taken into consideration [107].

Current treatments for HFREF have significantly ameliorated adverse symptoms and prolonged life. However, even with best current therapy mortality in HF remains high. This mandates a continuing search for new therapies in HF. Newer drugs that further target the RAAS pathway such as direct renin inhibitors, aliskiren, or the non-steroidal MRA, Finerenone, (BAY 94-8862) are currently under evaluation in clinical trials. With respect to more novel targets the NP have provided one path to new therapeutics. Their cardioprotective effects include attenuation of vasoconstriction, sodium retention, hypertrophy, fibrogenesis, and cell death [108,109]. Therapeutics designed to prolong or increase the bioactivity of NPs are under development. Neprilysin inhibition or infusions of synthesized natriuretic peptides or agonists are used to increase the level of peptide hormones known to provide cardioprotective effects [110,111]. In the most profound advance in HF therapeutics in the last 15 years, the combination of angiotensin receptor blockade and neprilysin inhibition is effective in HF [112]. A new drug, LCZ696, in a major clinical trial in HFREF significantly reduced death and hospital admissions in chronic HFREF [6]. Several other novel agents targeting various neurohormonal signaling cascades—to enhance cardiac contractility, vasodilation, as well as renal preservation, or to antagonize overdriven RAAS, SNS, and inflammatory mechanisms—are also currently undergoing various stages of clinical development [113,114]. Therapies based on urodilatin and cenderitide are designed to exert cardio- and renal-protective, as well as anti-fibrotic effects via activation of natriuretic peptide/cGMP signaling and have been shown to improve cardiac dysfunction in preclinical models [115,116]. Stimulators of soluble guanylate cyclase, such as Cinaciguat (BAY 58-2667), proposed to counteract desensitized nitric oxide-cGMP signaling in HF patients, have reduced cardiac overload and relieved dyspnea in phase II trials [117]. Treatment with the recombinant form of the relaxin-2 peptide hormone, serelaxin, enhances renal filtration and reduces angiotensin II-induced vasoconstriction by activating G protein mediated phosphorylation/nitric oxide signaling cascades [118]. Infusion of urocortins (particularly urocortin2) or of the cardiac growth factor, neuregulin 1, increases cardiac output in HF. Urocortin and neuregulin activate PI3K/AKT/eNOS-mediated pathways and Erb-neuregulin signaling, respectively [119,120,121,122].

Lastly, several drugs that have yet to reach preclinical and/or clinical trials have potential in HF treatment. These include the Angiotensin II Type I receptor biased ligands, TRV120023 and TRV120027, which do not trigger the classical (unwanted) angiotensin 2 type 1 receptor actions but do activate a beneficial post-receptor pathway, have produced favorable results in animal models of HF. These biased ligands preserve renal function, reduce cardiac overload, and promote cardiac contractility [123,124]. Capadenoson, a partial adenosine A1 receptor agonist, improves ventricular function and impedes progression of adverse cardiac remodeling in a canine HF model [125]. All these developments herald an exciting era of new therapeutics in HF.

## 5.miRNAs in Heart Failure

The discovery and understanding of miRNAs has raised the possibility of using circulating miRNA as biomarkers in cardiovascular disease [126,127]. miRNA microarray profiling or quantitative PCR array studies have examined the miRNA profiles obtained from various HF platforms. Table 4, Table 5, Table 6, Table 7 and Table 8 lists candidate miRNAs with potential utility as biomarkers of HF compiled from 21 research articles published between 2008 and 2015. Approximately half of the studies (11 out of 21) first performed miRNA profiling using a relatively small cohort size in the discovery phase, and subsequently verified the identified miRNA entities using a larger number of subjects in the validation phase. These HF-related miRNA entities are grouped according to the sample matrix used for miRNA profiling, namely whole blood (Table 4), serum (Table 5), plasma (Table 6), cardiac tissues/biopsy (Table 7), and peripheral blood mononuclear cell (PBMC) and buffy coat (Table 8). Each of these studies reported clusters of miRNAs that could distinguish HF from non-HF subjects and/or that could provide further differentiation between HFPEF from HFREF [2,128,129,130,131,132,133,134,135,136,137,138,139,140,141,142,143,144,145,146,147,148]. Little consensus on the HF miRNA signature exists across the 21 studies. The lack of agreement between studies is in jarring contrast to studies of NH such as NT-proBNP in HF. Contributing factors may include: variability in the phenotype, acuity or severity of HF, the appropriateness of controls, and small sample sizes. The background complexity of the pathophysiology of HF, which entails contributions (which vary in timing and severity) from cardiac, renal, vascular, pulmonary, adrenal, endocrine, haematological and biochemical perturbations, may also add to the potential for inter-cohort variation in miRNA profiles.

The information provided in the first two columns of the Table 4, Table 5, Table 6, Table 7 and Table 8 indicates that the 21 cohorts providing published miRNA data are heterogenous with respect to the etiology of HF. Ten studies have defined causes for HF. Two were focused on atrial fibrillation-induced HF (AF-HF). Three studies addressed HF secondary to acute myocardial infarction (AMI-HF) or other acute coronary syndromes (ACS-HF). Two reports focused on stable compensated dilated cardiomyopathy (DCM-HF) and myocarditis-induced DCM-HF. One paper reported on decompensated congestive HF secondary to DCM (DCM-CHF). A single report focused on ischemic cardiomyopathy-induced HF (ICM-HF) and one study on idiopathic cardiomyopathy induced HF (IDCM-HF). miRNA entities reported in the different studies were verified by using RT-PCR, and are reported as able to distinguish acute and/or chronic HF from control cases.

Among these HF-related miRNAs, four were consistently dysregulated in at least two study cohorts when compared with corresponding controls. miR-1 plasma levels were found up-regulated in patients with AMI-HF [133,144]. miR-195 levels were elevated in both DCM-HF left ventricle tissues and HF myocardial biopsy [142,148]. miR-30a serum levels were up-regulated in HF and HFREF [137,139], and miR-499 plasma levels were increased in acute HF and ACS-HF [135,144]. Additionally, differential expression of miR-126 relative to control was found in three sample matrixes including plasma, serum and in circulating endothelial progenitor cells. Interestingly, several miRNAs, such as miR-1 and miR-21, were observed to be differentially expressed in the circulation as well as in cardiac tissues from HF patients, suggesting a possible correlation between the miRNAs observed in circulation and events in cardiac tissue. Among all 71 reported miRNAs 13: miR-1, -124-3p, -126, -150, -195, -21, -210, -30a, -342-3p, -423-5p, -499-5p, -622 and -92a, were found to be differentially regulated in more than one HF cohort and are thus of special interest. Some of these appear to target important genes that are involved in cardiac remodeling. For example, miR-1 and miR-30a are reported to play roles in cardiac hypertrophy and apoptosis [149,150,151,152]. miR-21 targets key molecules in the signaling pathways that govern cardiac fibrosis, hypertrophy and apoptosis [150,153,154]. miR-195, miR-499-5p, and miR-92a target genes involved in apoptosis signaling [155,156,157]. The dysregulation of these 13 miRNAs across HF platforms may identify key elements of HF pathogenesis and warrants further investigation to identify their downstream functions/target genes.

### Putative miRNA Targets and Neurohormone

NHs are pivotal to cardiovascular homeostasis and play a key role in the pathogenesis of HF. Although differential expression patterns of miRNAs in HF have been reported (Table 4, Table 5, Table 6, Table 7 and Table 8), there is little information available on miRNAs with possible regulatory roles in NH signaling. Sucharov and colleagues demonstrated that miR-100 and miR-133b are up- and down-regulated, respectively, in idiopathic and ischemic cardiomyopathies. In neonatal rat cardiac ventricular myocytes miR-100 altered gene expression of adult isoforms of cardiac genes. MiR-133b has important functions in regulation of cardiomyocyte hypertrophy [148]. However, specific candidate gene targets of these regulatory miRNAs have not been identified. Recent work reported by our group pointed to a possible gene target for miR-100 with clinical relevance in HF. We applied multiple prediction algorithms to identify the gene target of miR-100 and found complementarity to the 3′UTR of natriuretic peptide receptor 3 (NPR3). We verified the miR-100-NPR3 interaction by loss-of-function and miR-NPR3 luciferase reporter assays. We demonstrated in cellular models the negative regulatory effect of miR-100 on NPR3 expression. This was also observed in rat myocardial infarct tissue. These data suggest elevation of miR-100 observed in HF patients may reflect a compensatory mechanism to attenuate the expression of NPR3 and thus prolong the half-life of circulating and tissue based natriuretic peptides [158]. Another HF-related miRNA, miR-125a/b-5p, is negatively associated with endothelin-1 levels in the aortae of stroke-prone hypertensive rats. Mir-125a/b-5p targets the 3′UTR of prepro-endothelin-1 and modulates the expression of endothelin in endothelial cells [159].

Indirect evidence also supports the role of HF-related miRNAs in modulating gene expression in NH signaling. One study using *in silico* and gene expression analysis suggested several genes associated with angiotensin II signaling in cardiac fibroblasts were regulated by miR-132/212 [160]. In vascular smooth muscle cells, miR-21 cross talks with ANP and nitric oxide via modulation of downstream cGMP signaling [161]. Other works demonstrate that antagonism between β1-adrenergic receptor stimulation and AKT survival signaling is mediated by miR-199a-5p [162]. Several recent studies have reported a number of newly discovered miRNAs, which may negatively regulate NH activity. Evidence from luciferase reporter assays demonstrated that miR-155 interacts with the 3′UTR of the angiotensin II type I receptor (AGTR1) transcript [163]. miR-425 interacts with the 3′UTR of ANP and may down regulate ANP production. AntagomiR-mediated attenuation of miR-425 may be a potential therapeutic approach for HF [164]. Finally, Maharjan and co-workers demonstrated that miR-766 downregulated the expression of the human aldosterone synthase gene, CYP11B2, by binding to the 735G-allele of the 3′UTR of CYP11B2 transcripts with a subsequently reduction in blood pressure [165].

We sought to uncover potential associations between HF-related miRNA entities and NH signaling cascades and thus identify potential therapeutic targets. We applied three miRNA target prediction algorithms, TargetScan v7.0 [166,167], miRDB [168,169] and miRanda [170,171] for *in silico* analyses and found putative target sites towards the 3′UTR of NH for the 71 HF related miRNA entities (Table 9). Interestingly, 62 out of the 71 miRNAs are predicted to target at least one NH gene. Further comparisons revealed a high percentage of miRNAs target NH receptors such as Angiotensin II receptors (AGTRs), Endothelin receptors (EDNRs), Corticotropin-releasing factor receptors (CRHR2), Mineralocorticoid receptor/Nuclear receptor subfamily 3 group C member 2 (NR3C2), and Natriuretic peptide receptors (NPRs), suggesting miRNA modulates NH signaling cascades in HF in part by attenuating the expression of the cognate receptors.

Algorithm-predicted interactions between miRNAs and putative target genes do not necessarily reflect true biologically relevant miRNA-target pairs. MiRNA-target prediction algorithms are based on either simple discriminative rules derived from experimental observations of important target recognition features, for example, matching of complementary seed region between miRNA-target mRNA (Targetscan, miRanda) or on a data-driven approach where a statistical model is built from training data and predictions are made based on the models (miRDB). The major challenge in target prediction arises from the fact that base pairing between the miRNA and its target is almost always imperfect where pairing of as little as eight base pairs have been shown to produce a regulatory effect [172,173]. In addition, although base pairing occurs mostly in the 3′UTR of target genes, a few cases of pairing with the 5′UTR and coding regions have also been observed. A further confounding factor in target prediction stems from the observations that the 3′UTR of mRNAs may contain multiple binding sites thereby increasing the possibility of a variety of miRNA binding partners. Thus, *in vitro* and/or *in vivo* experimental confirmation of genuine interactions between NH signaling gene targets and miRNAs is crucial. Nonetheless, the list of predicted gene targets in Table 9 points to potential discoveries on the roles of miRNAs in cardiovascular biology and pathophysiology, and provides a useful starting point for designing oligonucleotide-based mimics for *in vivo* miRNA manipulation of NH signaling.

## 6. Challenges of MicroRNA Research in Heart Failure

### 6.1. Consistent miRNA Profiles in Heart Failure Are Yet to Be Identified

Associations of miRNAs with various cardiovascular diseases including HF have been revealed in numerous clinical cohorts. It is known that miRNAs participate in regulation of most genes. Thus the distinct miRNA profiles observed in diseased tissues as well as in the circulation will in part reflect the underlying molecular pathology of the disease. MiRNAs have potential as diagnostic, prognostic and theragnostic biomarkers, as well as constituting rational therapeutic targets [25]. The compiled miRNA entities identified from 21 HF cohorts did not reveal any consistent “signature” HF miRNA profile common to all or even most studies (Table 4, Table 5, Table 6, Table 7 and Table 8). Although this unfavorable observation is somewhat surprising, it is however not implausible in view of the etiological complexity and dynamic nature of HF. It is also important to bear in mind that many non-cardiac sources of variation can also contribute to the disparate miRNA entities identified in the various studies, such as the small cohort size, differences in gender ratio, ethnicity, underlying co-morbidities, clinical criteria for patient recruitment, and differences in the genomic profiling technologies used. Currently, several groups including ours are making concerted efforts to minimize sources of variation arising from sampling and methodologic issues in miRNA profiling by using large HF cohorts with well-defined inclusion/exclusion criteria for recruitment, and applying the most well-established gene expression platform, quantitative PCR. Discriminative miRNA(s) signatures or miRNA clusters for HF diagnosis and risk stratification may become available in the near future from these large cohort studies. Results from multiple global miRNA analyses can provide an unbiased approach in applying miRNA profiles for distinguishing HF sub-types. In addition, further refinement of algorithms for miRNA-mRNA target prediction will be useful for exploring the underlying pathological implications of HF-related miRNA entities.

In summary, disturbances in NH signaling are intrinsic to the pathogenesis of HF. In this review, we report from *in silico* analyses the previously unrecognized fact that a high percentage of published HF-related miRNAs have putative target sites on the 3′UTRs of NH and their receptors. Although validation of miRNA targets requires experimental verification, our computer-based approach allows preliminary identification of novel miRNA signatures whereby HF-related miRNAs and their integrated regulatory role in NH signaling in HF can be hypothesized and pursued in contrast to focusing on individual miRNA entities in isolation. A comprehensive and accurate picture of HF-related miRNA targets will require combining information from miRNA studies with data from next generation sequencing and proteomic profiling.

### 6.2. Obstacles to miRNA Therapy in Cardiovascular Diseases

In the post-genomics era, a comprehensive understanding of molecular variations, such as the miRNA profiles in diseased and non-diseased states, and subsequent development of personalized medicine is no longer in the realm of science fiction, but has become a plausible and exciting goal for research. The discovery of miRNA signatures for particular adverse biological events, such as viral replication in liver and tumor growth has led to the development of several miRNA-based therapies [174,175]. After years of intensive pre-clinical studies, miRNA therapies that target miR-122 and miR-34 for hepatitis C and cancer treatment, respectively, have led to the successful launch of clinical trials, namely the “Miravirsen study in null responder to pegylated interferon alpha plus ribavirin subjects with chronic Hepatitis C (NCT01727934)” and “A multicenter phase I study of MRX34, miRNA micr-RX34 liposomal Injection (NCT01829971)” [18,176]. Results from these miRNA clinical trials are eagerly awaited. Such clinical trials, if positive, will inspire and encourage more concerted efforts in miRNA research directed towards other human diseases.

In cardiovascular diseases, such as HF, several critical obstacles, including targeting specificity and delivery efficiency, remain significant challenges yet to be overcome. It is known that miRNAs can elicit synergistic effects in fine-tuning the expression of specific target genes in conjunction with other miRNAs. A growing body of evidence also indicates that miRNAs often form functional clusters in modulating the expressions of multiple genes within integrated signaling networks [21,177]. While the multigene-targeting capability of a miRNA species can be used to great advantage for treating complex diseases, such as HF where disturbances of more than one gene is involved, such a functional ability can also contribute to off-target effects that lead to unwanted consequences in normal tissues or organs.

For *in vivo* delivery of miRNA mimics or inhibitors, current technologies and principles are mostly adopted from interference RNA gene therapy. Viable miRNA therapies rely on chemical modifications of appropriately designed miRNA and antisense-miRNA molecules to enhance stability and improve specificity of binding. Synthetic or expression vector-based systems may be used as effective carriers to deliver the oligonucleotides to target sites. Various cationic polymeric and liposomal delivery vehicles are common synthetic methods used [178]. Both methods are designed to stabilize the negatively charged synthetic oligonucleotides and protect them from degradation *in vivo*. However, liposomal and polymeric delivery vehicles administered via intravenous injection tend to accumulate in solid tumors and in organs such as liver and spleen, but not within the cardiovascular system. Hence, alternative methods such as direct myocardial injection and improved homing vehicles conjugated to cardiovascular surface markers or pH low insertion peptide (pHLIP) have been used to increase delivery efficiency to the target cardiac tissues [179,180]. For HF treatment, as for other conditions, the development of a non-invasive and high efficacy tissue-specific delivery method is highly desirable.

Our understanding of the roles of miRNAs in signaling pathways is growing rapidly. More and more studies indicate unique miRNA patterns in disease states that may serve as diagnostic and prognostic biomarkers, as well as therapeutic targets. The major challenge to be resolved in miRNA research in complex diseases like HF, is accurate identification of target miRNAs from the huge amount of data generated by different profiling methods for diagnostic, prognostic and therapeutic applications. By reviewing the HF-related miRNA entities found in published works and exploring the possible interaction between miRNAs and NH we aim to identify miRNAs signatures specific to HF and exploit this knowledge to seek pathways to new therapies.

## Figures and Tables

**Table 1:** Risk factors for heart failure.

Established Risk Factors	Increased HF Risk	Life Style Factors
Coronary artery disease	Higher serum creatinine	Obesity
Hypertension	Lower FEV1:FVC ratios	Smoking
Diabetes mellitus	Lower hemogloblin concentrations	Lack of exercise
Atrial fibrillation		Excessive alcohol consumption
Valvular heart disease		
Dilated cardiomyopathy *		

* Dilated cardiomyopathy includes: Familial cardiomyopathy; Endocrine and Metabolic cardiomyopathy; Toxic cardiomyopathy; Tachycardia induced cardiomyopathy, Inflammation induced cardiomyopathy (post-myocarditis); Peripartum cardiomyopathy; Iron overload induced cardiomyopathy; Amyloidosis; Cardiac Sarcoidosis; Stress induced cardiomyopathy.

**Table 2:** Neurohormones in the cardiovascular system.

Neurohormone	Receptor	Cardiovascular Functions	Pathological Effects
Catecholamines epinephrine and norepinephrine [75,76]	α-AR β-AR	Activation of α-AR produces vasoconstriction effect. Activation of β-AR procures myocardial contraction (both inotropic and chronotropic), and vasodilation effects.	Arrhythmias, cardiomyopathy, and sudden death
Renin		Renin cleaves angiotensinogen into angiotensin I.	Same as Angiotensin II
Angiotensin II [71,77]	AGTR1, AGTR2	Angiotensin I then converted by angiotensin-converting enzyme to angiotensin II. Activation of AGTRs produces vasoconstriction effect, it also stimulates the SNS and increases the secretion of aldosterone and subsequently leads to production of arginine vasopressin.	Hypertrophy of the myocardium and cardiac remodeling
Aldosterone [77]	NR3C2	Activation of NR3C2 leads to sodium retention, potassium excretion and increase blood pressure.	Cardiac fibrosis and remodeling
Arginine vasopressin [71,77]	V1R V2R	Activation of V1R leads to vasoconstriction effect. Stimulation of V2R leads to retention of water, and antidiuretic effect.	Hyponatremia and antidiuresis
Endothelin [71,77,78]	EDNRA EDNRB	Activation of EDNRA causes vasoconstriction, while activation of EDNRB leads to vasodilation.	Hypertrophy. Systemic and renal vasoconstriction
ANP [79,80]	NPR1 NPR3	Activation of NPR1 leads to vasodilation, diuresis, natriuresis. It also suppresses RAAS, SNS, and have an anti-hypertrophic effect.	Hypotension
BNP [79,80]	NPR1 NPR3	Activation of NPR1 leads to vasodilation, diuresis, natriuresis. It also suppresses RAAS, SNS, and have an anti-hypertrophic effect.	Hypotension
CNP [79]	NPR2 NPR3	Activation of NPR1 leads to vasodilation, diuresis, natriuresis and have an anti-proliferative effect.	
ADM [5,81]	GPR-182	Vasodilatation with inotropism and natriuresis	
Urocortins [82,83,84]	CRHRs	Positive inotropic and chronotropic effects, arterial and venous dilatation	

ANP, Atrial natriuretic peptide; BNP, Brain natriuretic peptide; CNP, C type natriuretic peptide; ADM, Adrenomedullins; α-AR, α adrenergic receptor; β-AR, β adrenergic receptors; AGTR1, Angiotensin II receptor type 1; AGTR2, Angiotensin II receptor type 2; NR3C2, Mineralocorticoid receptor/Nuclear receptor subfamily 3 group C member 2; V1R, Vasopressin type 1 receptor; V2R, Vasopressin type 2 receptor; EDNRA, Endothelin receptor type A; EDNRB, Endothelin receptor type B; NPR1, Natriuretic peptide receptor type 1; NPR2, Natriuretic peptide receptor type 2; NPR3, Natriuretic peptide receptor type 3; GPR-182, G-protein coupled receptor 182; CRHRs, Corticotropin-releasing factor receptors.

**Table 3:** HF signature signaling cascades and the key factors that have been proposed to be the HF biomarkers.

HF Signaling Cascades	Biomarkers for HF
Neurohormonal activation	Norepinephrine, Renin activity, Angiotensin, Aldosterone, Arginine-Vasopressin
Myocardial overload	BNP, NT-proBNP, MR-proANP, MR-proADM
Cardiac injury	Troponin T, Troponin I, LOX-1, GDF-15
Cardiac remodeling	IL-6, TNFα, CRP, MMP, Galectin-3, Soluble ST2

BNP, brain natriuretic peptide; NT-proBNP, N-terminal pro brain natriuretic peptide; MR-proANP, Mid-region pro atrial natriuretic peptide; MR-proADM, Mid-region pro adrenomedullin; LOX-1, lectin-like oxidized low density lipoproteins receptor-1; GDF-15, growth differentiation factor-15; TNFα, tumor necrosis factor alpha; CRP, C-reactive protein; MMP, matrix metalloproteinases.

**Table 4:** Summary of reported miRNAs as HF biomarkers in whole blood samples.

Study Cohort		NT-proBNP, LVEF and Other Criteria	Platform	miRNA Identified	Diagnostic Potential	Reference
Discovery	Validation					
Whole blood and plasma, no-HF (*n* = 28), HFREF (*n* = 39) and HFPEF (*n* = 19)	Plasma from no-HF (*n* = 30), HFREF (*n* = 30) and HFPEF (*n* = 30)	NT-proBNP: 3086 ± 421 pg/mL; HFPEF: LVEF ≥ 50%, HFREF: LVEF ≤ 40%	miRNA microarray and RT-PCR	miR-1233, -183-3p, -190a, -193b-3p, -193b-5p, -211-5p, -494, and -671-5p	HF	Wong *et al.* [129]
				miR-125a-5p, -183-3p, -193b-3p, -211-5p, -494, -638, and -671-5p	HFREF	
				miR-1233, -183-3p, -190a, -193b-3p, -193b-5p, and -545-5p	HFPEF	
				miR-125a-5p, -190a, -550a-5p, and -638	HFREF *vs.* HFPEF	
Whole blood, from control (*n* = 39) and HFREF (*n* = 53)	Serum from controls (*n* = 8) and HFREF (*n* = 14)	NT-proBNP: 2399 ± 3395 ng/L, HFREF: LVEF < 50%	miRNA microarray and RT-PCR	miR-200b-5p, miR-622, miR-1228-5p	HFREF	Vogel *et al.* [2]

AF, Atrial Fibrillation; HF, Heart Failure; HFREF, Heart failure with reduced ejection fraction; HFPEF, Heart failure with preserved ejection fraction; LVEF, Left ventricular ejection fraction; BNP, brain natriuretic peptide; NT-proBNP, N-terminal pro brain natriuretic peptide; *n*, sample size.

**Table 5:** Summary of reported miRNAs as HF biomarkers in serum samples.

Study Cohort		NT-proBNP, LVEF and Other Criteria	Platform	miRNA Identified	Diagnostic Potential	Reference
Discovery	Validation					
Serum from control (*n* = 32), AF (*n* = 35), HF (*n* = 32), HF-AF (*n* = 36)		NYHA class III, IV, Log(NT-proBNP): 4.07 ± 0.51, LVEF: 48.32% ± 6.00%	RT-PCR	miR-126	Severity of AF and HF	Wei *et al.* [141]
Serum pooled from *n* = 15 per group in no-HF, HFREF and HFPEF	Serum from *n* = 75 per group in no-HF, HFREF and HFPEF	HFPEF: NYHA class IV, LVEF ≥ 50%, BNP: 215 (126–353) pg/mL, HFREF: LVEF < 50%, BNP: 139 (71–254) pg/mL	qPCR array, RT-PCR	miR-30c, miR-146a, miR-221, miR-328, miR-375	HF and HFREF *vs.* HFPEF	Watson *et al.* [128]
Platelets and serum from control (*n* = 35), HF (*n* = 26) and AF-HF (*n* = 15)		HF: LVEF < 40%, NYHA class I-IV, BNP: 147 (47–416) pg/mL	RT-PCR	miR-150	AF-HF	Goren *et al.* [140]
Serum from control (*n* = 7) and HF (*n* = 7)	Serum from control (*n* = 65) and HF (*n* = 21)	Patients with acute stage of AMI onset (Killip class > II) developed HF	qPCR array, RT-PCR	miR-192, miR-194, miR-34a	HF after AMI	Matsumoto *et al.* [136]
Serum from 2 pooled samples of control (*n* = 6) and HF (*n* = 6)	Serum from control (*n* = 30) and HF (*n* = 30)	Chronic stable class C HFREF with LVEF < 40%, BNP: 180 (98–276) pg/mL	qPCR array, RT-PCR	miR-423-5p, -320a, -22, -92b, -17, -532-3p, -92a, -30a, -21, -101	HFREF	Goren *et al.* [137]
Serum from control (*n* = 18), HF (*n* = 22)		NYHA class III, IV, pro-BNP ≥ 1000 ng/L	RT-PCR	miR-210, miR-30a	HF	Zhao *et al.* [139]

AMI, Acute myocardial infarction.

**Table 6:** Summary of reported miRNAs as HF biomarkers in plasma samples.

Study Cohort		NT-proBNP, LVEF and Other Criteria	Platform	miRNA Identified	Diagnostic Potential	Reference
Discovery	Validation					
Plasma from AMI patients (*n* = 49) with various EF		AMI patient: cardiac troponin, creatine kinase-MB, Q-waves and ST-segment elevation	RT-PCR	miR-1	HF after AMI	Zhang *et al.* [133]
Plasma from control (*n* = 20) and HF (*n* = 33)		Framingham criteria, NT-pro-BNP > 200 pmol/L	RT-PCR	miR-499, miR-122	Acute HF	Corsten *et al.* [135]
Plasma from control (*n* = 12), HF (*n* = 12)	Plasma from control (*n* = 39), HF (*n* = 30)	Framingham criteria, NT-proBNP > 1000 ng/L	miRNA microarray RT-PCR	miR-423-5p, -18b-3p, -129-5p, -1254, -675, -622	Acute HF	Tijsen *et al.* [131]
Plasma from ACS (*n* = 424)		Coronary artery bypass grafting patients and ACS patient with STEMI and NSTEMI	RT-PCR	miR-1, miR-208, miR-499-5p	HF after MI	Gidlöf *et al.* [144]
Plasma from control (*n* = 14) and HF (*n* = 32)	Plasma from HF (*n* = 44) and control (*n* = 15)	Discovery cohort: HFREF: 27.3 ± 9.0, HFPEF: 57.8 ± 7.0, NT-proBNP:460.8 (141.3–2511.9) pmol/L, Validation cohort: HFREF:27.0 ± 7.7, HFPEF:62.0 ± 6.4, NT-proBNP: 493.28 (25.7–3801.9) pmol/L	qPCR array RT-PCR	miR-185, miR-103, miR-142-3p, miR-30b, miR-342-3p, miR-150	Acute HF	Ellis *et al.* [130]
Plasma from HF (*n* = 8) and control (*n* = 3)	Control (*n* = 17), HF (NYHA II) (*n* = 17), NYHA III (*n* = 6) and NYHA IV (*n* = 10)	NYHA class II–IV	miRNA microarray RT-PCR	miR-126	HF	Fukushima *et al.* [134]

ACS, acute coronary syndrome; STEMI, ST segment elevation myocardial infarction; NSTEMI, non-STEMI:NYHA, New York Heart Association (NYHA) Functional Classification.

**Table 7:** Summary of reported miRNAs as HF biomarkers in cardiac tissues/biopsy samples.

Study Cohort		NT-proBNP, LVEF and Other Criteria	Platform	miRNA Identified	Diagnostic Potential	Reference
Discovery	Validation					
Myocardial biopsy from control (*n* = 17) and HF (*n* = 17)		LVEF mean: 30%, HF due to myocarditis or DCM	RT-PCR	miR-1, -21, -23, -29, -130, -195, -199	HF	Lai *et al.* [142]
LV Tissue from non-failing (*n* = 10) and DCM (*n* = 30)	LV Tissue from non-failing (*n* = 10) and DCM (*n* = 20)	DCM with EF 15% ± 1%	miRNA microarray RT-PCR	miR-1, -29b, -7, -378, -214, -342, -145, -125b, -181b	HF	Naga Prasad *et al.* [147]
LV Tissue Non-failing (*n* = 6), IDCM (*n* = 5), Ischemic DCM (*n* = 5)		IDC and ISC patients	miRNA microarray RT-PCR	miR-100, miR-195, miR-92, miR-133b	HF	Sucharov *et al.* [148]

DCM, stable compensated dilated cardiomyopathy; IDC, Idiopathic cardiomyopathy; ISC, ischemic patients.

**Table 8:** Summary of reported miRNA as HF biomarker in peripheral blood mononuclear cells (PBMC) endothelial progenitor cells (EPC) and buffy coat samples.

Study Cohort		NT-proBNP, LVEF and Other Criteria	Platform	miRNA Identified	Diagnostic Potential	Reference
Discovery	Validation					
Mononuclear from control (*n* = 6), NYHA II (*n* = 8) and NYHA III, IV (*n* = 5)		NYHA class II–IV	RT-PCR	miR-210	HF	Endo *et al.* [132]
Buffy coat HFPEF (*n* = 8), DCM (*n* = 10), DCM-CHF (*n* = 13), Control (*n* = 8)		HFPEF with mean LVEF 61.13 and mean BNP 353.99 pg/mL, DCM-HF with mean LVEF 19.23 and BNP 2247 pg/mL	miRNA microarray RT-PCR	miR-454, miR-500, miR-1246,	HFPEF	Nair *et al.* [145]
				miR-142-3p, miR-124-5p	DCM-HF	
PBMC from control (*n* = 9) and HF (*n* = 15)	PBMC from control (*n* = 19) and HF (*n* = 34)	NYHA class III/IV with mean LVEF ≤ 36%	RT-PCR	miR-139, miR-142-5p, miR-107	Chronic HF	Voellenkle *et al.* [146]
EPC from control (*n* = 10), ICM-HF (*n* = 10) and NICM-HF (*n* = 10)	EPC from control (*n* = 30), ICM-HF (*n* = 55) and NICM-HF (*n* = 51)	NYHA class III, IV	qPCR array RT-PCR	miR-126, miR-508-5p	HF	Qiang *et al.* [143]

DCM-CHF, decompensated congestive HF secondary to DCM; ICM, ischemic cardiomyopathy; EPC, Endothelial progenitor cells.

**Table 9:** Predicted neurohormones targets for 71 heart failure related miRNAs.

miRNA	Targetscan		miRDB	miRanda	
	Conserved	Poorly Conserved	Gene (Target Score *)	Good mirSVR Score and Conserved	Non-Good mirSVR Score and Conserved
miR-1	–	AGTR1	–	AGTR1, EDNRB	AGT, ACE, EDN1, EDNRA
miR-100	–	–	–	–	NPR3
miR-101-3p	–	ACE	–	AGTR2, CALCRL, EDN1, EDNRB, NR3C2	AGT, CALCRL, EDN1
miR-103a-3p	CRHR2	AGT, AGTR1, NPPA	–	REN	AGT, CRHR1, UCN2, NR3C2, NPR2, NPPA, EDNRA, EDN1, ATP6AP2, ACE, AGTR2, AGTR1
miR-107	CRHR1	AGT, AGTR1, NPPA	–	REN	AGT, AGTR1, AGTR2,ACE, ATP6AP2, EDN1, EDNRA, NPPA, NPR2, NR3C2, UCN2, CRHR1
miR-122	–	–	–	ATP6AP2, EDN1, NPR3, CRHR1	ACE, ATP6AP2, EDNRA, CRHR1, CRHR2, NR3C2, CYP11B2
miR-1228-5p	–	–	–	–	–
miR-1233-3p	CRHR2	–	–	–	–
miR-124-5p	–	–	–	AGTR1, EDNRB, NR3C2	ACE, EDN1, NPR1, CYP11B2, NR3C2, CRHR1
miR-1246	–	–	–	–	–
miR-1254	–	ACE, NPR1, CYP11B2	NPR3(63)	–	–
miR-125a-5p	CRHR2	ACE, CYP11B2	–	NPR3, CYP11B2	–
miR-125b-5p	CRHR1	ACE, CYP11B2	–	NPR3, CYP11B2	AGTR2, ACE, EDN1, EDNRA
miR-126-3p	–	–	–	–	–
miR-129-5p	–	AGT, NPR1, NPR2	NR3C2(84), AGTR1(76)	EDN1, EDNRA, EDNRB, NPR3, NR3C2	ACE, CALCRL, ATP6AP2, EDN1, EDNRA, EDNRB, NPR2, NPR3, AGT
miR-130a-3p	–	NPR1	EDN1(69)	ATP6AP2, EDN1, NR3C2	AGT, ACE, EDN1, EDNRA, NR3C2, CRHR1
miR-133b	–	–	ATP6AP2(54)	ATP6AP2, CRHR1	
miR-139-5p	–	NPPA	–	CALCRL, EDNRB, NPPA, NPR3, NR3C2	ACE, CALCRL, EDNRA, EDNRB, NR3C2
miR-142-3p	–	-	–	CALCRL, NR3C2	ACE, CALCRL, EDNRA, EDNRB, NR3C2
miR-142-5p	AGTR2	AGT, ACE	–	–	–
miR-145-5p	–	AGT	–	AGTR2, CALCRL	AGTR2, ACE, ATP6AP2, EDN1, EDNRB, CRHR1
miR-146a-5p	–	CRHR2, NPR1, CRHR2	–	CALCRL, EDNRB, NPR2, NPR3	–
miR-150-5p	CRHR2	GRP182, NPR1	–	ATP6AP2, EDNRB, NPR3	–
miR-17-5p	–	AGTR2, NPR1	–	AGTR2, ACE, NPR3	AGTR2, ACE, CALCRL, EDN1, EDNRA, EDNRB, NPR3, NR3C2
miR-181b-5p	–	AGT, AGTR1	ADM(74), CALCRL(56)	AGTR1, ADM, CALCRL, NPR3, ATP6AP2, EDNRB, NR3C2	AGT, ACE, EDRNA
miR-183-3p	–	AGTR1	–	–	–
miR-185-5p	CRHR2	ACE, NPR1, CYP11B2	–	CYP11B2	–
miR-18b-3p	–	CYP11B2	–	–	–
miR-190a	–	–	–	–	–
miR-192	–	–	–	NPR3	AGTR2, ACE, CALCRL, EDN1, UCN2, CRHR1
miR193b-3p	–	AGT, CYP11B2, CRHR2	–	EDN1	–
miR-193b-5p	–	NPR1	–	–	–
miR-194-5p	NPPA	–	EDN1(70)	EDN1, NPPA, NPR3	–
miR-195-5p	–	AGT, CYP11B2, CRHR2	–	AGTR2, NPR2, NPR3	–
miR-199a-5p	–	ACE	–	AGTR2, DNRA, EDNRB, UCN2	AGTR1, ACE, ATP6AP2, EDN1, EDNRA, CYP11B2, CRHR1, CRHR2
miR-200b-5p	–	AGTR1	–	–	–
miR-208a	AGTR2	–	–	ATP6AB2, EDNRB	AGTR1, CALCRL, UCN2
miR-21-5p	–	–	NPPB(69)	EDNRB	EDNRA, NPPA, NPPB
miR-210-5p	–	NPR1	–	CRHR2	NR3C2
miR-211-5p	–	–	NR3C2(86)	CALCRL, ATP6AP2, EDNRA, NPR3, NR3C2, CRHR2	ATP6AP2, EDN1, EDNRA, CRHR1, CRHR2
miR-214-3p	–	ACE, REN	–	AGTR1, CALCRL, REN, EDN1, EDNRB, CRHR1	EDN1, EDNRA, NPPA, NPR2, UCN2
miR-22-3p	–	AGT	–	ACE, EDNRA, NPR3, CRHR1	ACE, NPPA, NPR2, CYP11B2, NR3C2, CRHR1, CRHR2
miR-221-3p	–	ACE	–	NPR3, NR3C2	ACE, CALCRL, EDNRA, EDNRB, NPR2, NR3C2, CRHR1
miR-23a-3p	–	NPR1	NPR3(60)	AGTR2, CALCRL, EDNRB	ACE, ADM, CALCRL, EDN1, EDNRA, NR3C2
miR-29a-3p	–	–	–	EDNRB, NPPA, NPR3	AGTR1, ACE, EDNRB, CYP11B2, UCN2
miR-29b-3p	–	–	–	EDNRB, NPPA, NPR3	AGTR1, ACE, EDNRB, CYP11B2, UCN2
miR-30a-5p	–	–	EDNRA(54)	EDN1, EDNRA, EDNRB, NPR3	AGTR1, AGTR2, EDNRA, EDNRB, NR3C2
miR-30b-5p	–	–	EDNRA(54)	AGTR1, EDN1, EDNRA, EDNRB, NPR3	AGTR2, EDNRA, EDNRB, NR3C2
miR-30c-5p	–	–	EDNRA(54)	AGTR1, EDN1, EDNRA, EDNRB, NPR3	AGTR2, EDNRA, EDNRB, NR3C2
miR-320a	–	NPPB	–	EDNRA, NPPB, NPR3, NR3C2, EDNRA, NPR3, NR3C2	–
miR-328-3p	–	AGT, CYP11B2	–	UCN2	CRHR2
miR-342-3p	–	–	–	UCN2, CRHR2	–
miR-34a-5p	CRHR1	NPR1	UCN2(95), CRHR1(54)	AGTR1, EDNRB, NR3C2, UCN2, CRHR1	AGT, ACE, CALCRL, EDN1, EDNRA, EDNRB
miR-375	–	AGT, GTR1	–	ATP6AP2	AGT
miR-378a-5p	–	AGTR2, NPR1, CYP11B2	–	EDN1, CYP11B2, CRHR1	–
miR-423-5p	–	AGT, REN, CRHR2	CRHR2(56)	–	–
miR-454	–	NPR1	–	ATP6AP2, EDN1, NPR3, NR3C2	–
miR-494	–	–	–	AGTR1, END1, EDNRA, EDNRB, NPR3	–
miR-499-5p	–	–	–	CALCRL, ATP6AP2	–
miR-500a-5p	–	AGTR2	CALCRL(53)	–	–
miR-508-5p	–	ACE	–	–	NPR1
miR-532-3p	–	GPR182, CRHR2	NPR3(64)	–	–
miR-545-5p	–	AGTR1, NPPA	–	–	–
miR-550a-5p	–	GPR182, NPR1	–	NPR1	–
miR-622	–	AGT, NPPA, NPR1	–	–	–
miR-638	–	CYP11B2	–	–	–
miR-671-5p	–	ACE, CYP11B2, CRHR2	DN1(82)	–	–
miR-675	–	–	–	–	–
miR-7-5p	–	AGT, AGTR1	–	AGTR1, EDN1, NPR3	ACE, CALCRL, ATP6AP2, EDN1, EDNRA, CRHR1, CRHR2
miR-92a-3p	–	NPR1	–	AGTR2, ADM, EDNRB, NR3C2	AGTR1, CALCRL, EDNRA, EDNRB, NPR2, NR3C2
miR-92b-3p	–	NPR1	–	AGTR2, ADM, EDNRB, NR3C2	EDNRA

AGT, angiotensinogen (serpin peptidase inhibitor, clade A, member 8); AGTR1, angiotensin II receptor type 1; AGTR2, angiotensin II receptor type 2; ACE, angiotensin I converting enzyme (peptidyl-dipeptidase A); ADM, adrenomedullin; CALCRL, calcitonin gene-related peptide type 1 receptor; GPR182, G-protein coupled receptor 182; REN, renin; ATP6AP2, ATPase, H+ transporting, lysosomal accessory protein 2 (renin receptor); EDN1, endothelin 1; EDNRA, endothelin receptor type A; EDNRB, endothelin receptor type B; NPPA, natriuretic peptide A; NPPB, natriuretic peptide B; NPPC, natriuretic peptide C; NPR1, natriuretic peptide receptor A/guanylate cyclase A; NPR2, natriuretic peptide receptor B/guanylate cyclase B; NPR3, natriuretic peptide receptor C/guanylate cyclase C; CYP11B2, cytochrome P450, family 11, subfamily B, polypeptide 2 (aldosterone synthase); NR3C2, nuclear receptor subfamily 3, group C, member 2 (aldosterone receptor, mineralocorticoid receptor); UCN, urocortin; UCN2, urocortin2; CRHR1, corticotropin releasing hormone receptor 1; CRHR2, corticotropin releasing hormone receptor. * miRDB gene target scores represent the predicted scores assigned by the algorithm. The higher the score (>80), the more statistical confidence in the prediction result.
